# Overexpression of Platelet-Derived Growth Factor and Its Receptor Are Correlated with Oral Tumorigenesis and Poor Prognosis in Oral Squamous Cell Carcinoma

**DOI:** 10.3390/ijms21072360

**Published:** 2020-03-29

**Authors:** Li-Han Lin, Jiun-Sheng Lin, Cheng-Chieh Yang, Hui-Wen Cheng, Kuo-Wei Chang, Chung-Ji Liu

**Affiliations:** 1 Department of Medical Research, MacKay Memorial Hospital, Taipei 10449, Taiwan; volkdeskkimo@gmail.com (L.-H.L.); amycheng.b784@mmh.org.tw (H.-W.C.); 2 Department of Oral and Maxillofacial Surgery, MacKay Memorial Hospital, Taipei 10449, Taiwan; shengd85@yahoo.com.tw; 3 Institute of Oral Biology, School of Dentistry, National Yang-Ming University, Taipei 11221, Taiwan; 4 School of Dentistry, National Yang-Ming University, Taipei 11221, Taiwan; yangcc1124@gmail.com; 5 Department of Stomatology, Veterans General Hospital, Taipei 11217, Taiwan; 6 Department of Stomatology, Medical Education and Research, Veterans General Hospital, Taipei 11217, Taiwan

**Keywords:** oral squamous cell carcinoma, platelet-derived growth factor, platelet-derived growth factor receptor, survival

## Abstract

Oral squamous cell carcinoma (OSCC) is a cancerous disease with poor prognosis. According to the statistics, the 5-year survival rate has not improved significantly over the past 20 years. The platelet-derived growth factor (PDGF) and its signaling pathway is a key regulator of angiogenesis and tumorigenesis. High level of PDGF and its receptor (PDGFR) have been reported in several types of malignancies. In this study, we investigated the relationship of the molecular expression levels of PDGF and PDGFR with clinicopathological parameters in OSCC. To this end, we measured the mRNA and protein levels of PDGF and PDGFR by real-time quantitative PCR (qRT-PCR), immunohistochemistry, and enzyme-linked immunosorbent assay (ELISA), respectively. We found positive correlations of the mRNA levels of PDGFA, PDGFB, and PDGFRB with lymph node metastasis and poor overall survival (OS). High expression of PDGF, PDGFRA, and PDGFRB were remarkably associated with lymph node metastasis and poor OS, as determined by immunohistochemistry. Preoperative serum levels of PDGF-AA and PDGF-BB had a positive correlation with preoperative platelet count. Elevated serum levels of PDGF-AA. PDGF-BB, and platelet count correlated with lymph node metastasis and an unfavorable outcome. In multivariate Cox regression analysis, PDGFA mRNA, PDGFB mRNA, PDGFRB mRNA, PDGF immunoexpression, PDGFRB immunoexpression, serum PDGF-AA, serum PDGF-BB, and platelet count emerged as significant independent prognostic factors for OS. In vitro, we found that elevated PDGF promotes colony formation, migration, and invasiveness of SAS and OECM-1 cancer cell lines. Our results suggest that the expression level of serum PDGF has the potential to become a useful diagnostic marker for the prognosis of OSCC. In addition, PDGFR should be considered as a potential therapeutic target for OSCC. Furthermore, research should be undertaken to elucidate the role of PDGF and PDGFR regarding the behavior of tumor cells in OSCC.

## 1. Introduction 

Oral squamous cell carcinoma (OSCC) is an extremely special type of cancer. It is one of the fastest growing malignant tumors in Taiwan. Although various surgical operation techniques, chemotherapy, and radiation therapy have led to great improvements, the survival rate of oral cancer has not decreased significantly. According to statistics from the Ministry of Health and Welfare, the average survival rate within 5 years after the diagnosis of oral cancer has not improved significantly over the last 20 years [1]. For patients with late-stage OSCC (stages III and IV), the treatments are not very effective, as there is still a high risk of local recurrence and poor survival [2,3]. Therefore, understanding the genetic features of OSCC is needed to help in the control of OSCC. Early diagnosis is extremely helpful, and thus a molecular tumor marker is desired for the planning of cancer treatment programs and tracking patients.

From the viewpoint of molecular genetics, the genes and actions considered to play a role in tumorigenesis include the loss of tumor suppression genes, abnormal behavior of activated oncogenes, and the loss of heterozygous alleles on chromosomes, such as epidermal growth factor receptor (EGFR), TP53, COX-2, and cyclin D1 [4,5]. In addition, cytokines or growth factors, such as vascular endothelial growth factor (VEGF), platelet-derived growth factor (PDGF), fibroblast growth factor (FGF), tumor necrosis factor α (TNF-α), and tumor growth factor β (TGF-β) are considered to be related to the increased aggressiveness and metastatic properties of OSCC [6]. For example, VEGF in particular is related to the angiogenesis of tumors and is an important biomarker of tumor invasiveness [7,8]. In addition, the overexpression of the VEGF gene is correlated with the prognosis of oral cancers [9]. PDGF is also a crucial factor for tumor growth, angiogenesis, and tumor survival [10]. 

Thrombocythemia is the presence of an abnormally high number of platelets in circulating blood and can potentially result in various diseases. The functions of platelets include being the energy source of tumors, providing growth factors to tumors, and being the key element of cancer growth and metastasis [11]. In the relevant literature, the apparent increase in platelet number has been described for many malignant tumors, such as lung, uterine, esophageal, breast, stomach, kidney, and colorectal cancer, proving the association between thrombocythemia and cancers [12,13,14,15]. Dr. Lu found that patients with oral cancer exhibit notable increases in platelet count, particularly those with larger tumors or lymph node metastases [16]. However, the actual mechanisms of thrombocythemia in malignant tumors still are not clear. In the last few years, the primary role of thrombocythemia was found to be a potential humoral mediator. In some related studies on malignant tumors and thrombocythemia cytokines, the host’s immune system has been found to secrete considerable amounts of cytokines with an accompanying increase in platelet count when resisting tumor cells. For example, IL-6, IL-1, VEGF, PDGF, macrophage colony stimulating factor (M-CSF), and TNF-α have been found [17,18]. Other studies discovered that the increased expression of VEGF, TGF-α, and PDGF facilitates angiogenesis [19,20,21]. In tumor formation, angiogenesis is a critical mechanism in which growth factors including PDGF are playing a role [21]. PDGF is involved in the control of the cell cycle and of apoptosis, and it has been shown that the gene expression of PDGF and its receptors (PDGFR) are related to various diseases and cancerous processes [22]. 

PDGF is produced by platelets and stored in their α granules. It is also secreted by epithelial and mesenchymal cells [23,24,25]. The PDGF family consists of four members (PDGFA, PDGFB, PDGFC, and PDGFD), which form homo- and heterodimers [22]. The PDGFR receptors are encoded by two genes: PDGFRA and PDGFRB. The functional receptors are composed of homo- or heterodimers from α and β chains (αα, αβ, and ββ). When PDGFR is bound by its ligands, intracellular tyrosine kinases are activated and transduce the signal further into the cell. All PDGF ligands form homodimers through disulfide bonds except PDGFA and PDGFB, which form heterodimers [26,27]. The different ligands (including AA, BB, AB, CC, and DD) differ in their affinity for the different receptors (αα, αβ, and ββ) and activate different types of downstream signaling pathways [22]. PDGFR is known to control growth, cell movement, angiogenesis, and embryo development. PDGFRA knock-out mice have multiple developmental abnormalities, including defects of the lungs, the skeleton, the testes and the central nervous system [22,27,28]. In other studies, PDGF has been found to influence chemotaxis, movement, survival, apoptosis, and transformation of cells [22,29,30]. The overexpression of PDGFR is related to the generation of various human tumors, such as glioma, neurofibroma, prostate cancer, ovarian cancer, and non-small-cell lung carcinoma [31,32,33]. PDGF also facilitates angiogenesis and development of cancer-associated fibroblasts, which directly or indirectly influences the generation of tumors [34,35]. Furthermore, PDGF is involved in gene amplification and overexpression in various cancers [32,33,36]. Thus far, studies on PDGF and PDGFR gene expression in light of OSCC are extremely rare. The extent to which PDGF impacts OSCC pathogenesis and its clinicopathological features are relatively unclear. In this study, we investigated the prognostic significance of PDGF and PDGFR expression and of preoperative serum PDGF levels in respect to different clinicopathological features in a long-term follow-up of patients with OSCC. 

## 2. Results

### 2.1. PDGF and PDGFR mRNA are Upregulated in OSCC Tissues

**Figure 1:**
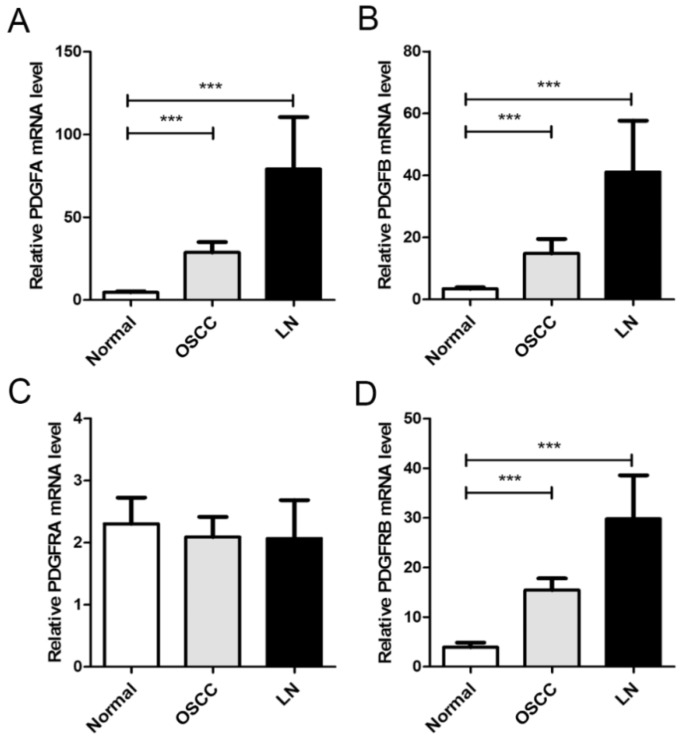
mRNA expression profiles of matched non-cancerous oral mucosa, cancer, and lymph node metastatic tissues. Histograms showing the mRNA levels of PDGFA (**A**), PDGFB (**B**), PDGFRA (**C**), and PDGFRB (**D**) in matched non-cancerous oral mucosa, cancer, and lymph node metastatic tissues. The relative mRNA expression levels were calculated using the 2^−^^ΔΔ^^Ct^ method. ***, *p* < 0.001

We first measured the mRNA levels of PDGF and PDGFR in matched normal, cancerous, and lymph node metastatic tissues. To this end, we collected samples of 126 OSCC tissues, matching non-cancerous oral mucosa, and 25 samples with matched lymph node metastatic tissue. Analysis by qRT-PCR demonstrated upregulation of PDGFA mRNA expression in 84.1% of OSCC tumors (106/126), relative to the non-cancerous matched oral mucosa, followed by PDGFB (71.4%, 90/126) and PDGFRB (81.0%, 102/126; Figure 1). PDGFA (88.0%, 22/25), PDGFB (80.0%, 20/25), and PDGFRB (84.0%, 21/25) had increased mRNA expression in metastatic lymph node tissue, compared to non-cancerous matched oral mucosa. No significant differences were observed in the mRNA expression levels of PDGFRA. The elevated mRNA levels of PDGFA and PDGFB correlated significantly with lymph node metastasis (*p* = 0.002 and *p* = 0.011, respectively; Table 1). Furthermore, increased levels of PDGFRB correlated significantly with lymph node metastasis (*p* = 0.026) and advanced TNM stage (*p* = 0.045). Only a marginally significant correlation was found between PDGFRA mRNA and lymph node metastasis (*p* = 0.064). 

### 2.2. Immunohistochemical Expression of PDGF and PDGFR in OSCC Tissues

**Figure 2:**
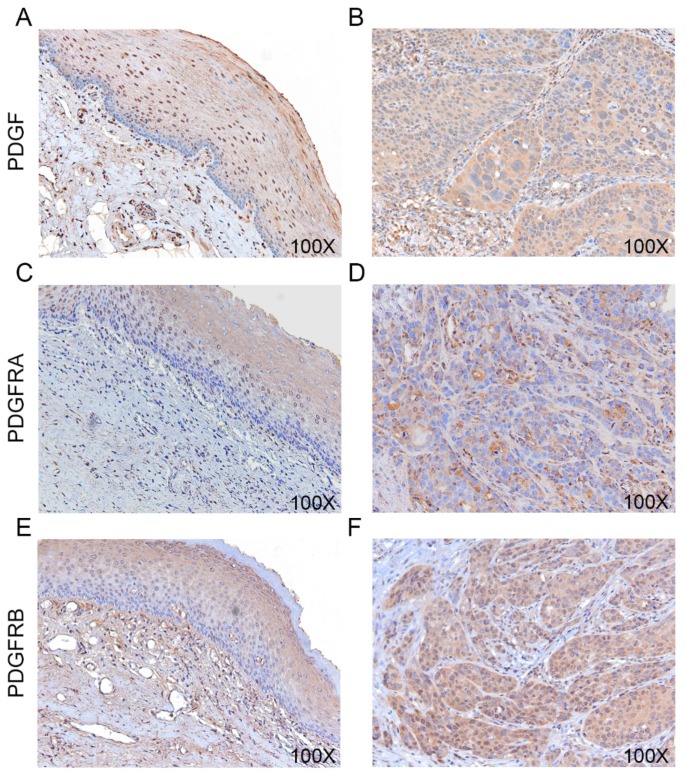
Immunohistochemical staining in OSCC. (**A**,**B**) Immunohistochemistry of PDGF in adjacent normal looking mucosa (A) and OSCC tumors (B). (**C**,**D**) PDGFRA immunoexpression. (**E**,**F**) PDGFRB immunoexpression. All IHC images were photographed at 100× magnification.

A gradual increase in PDGF, PDGFRA, and PDGFRB staining was apparent, progressing from normal-appearing oral epithelium to covering epithelium. The strongest staining was observed in invasive tumor cells. PDGF and PDGFRB immunoreactivity was present in both the cytosol and the nucleus (Figure 2B,F) while the PDGFRA immunoreactivity was present mainly in the cytosol (Figure 2D). A total of 55.6% of the tumors (35/63) showed intensive PDGF staining, 42.9% (27/63) had high PDGFRA immunoreactivity, and 41.3% (26/63) had high PDGFRB immunoreactivity (Table 2). The high expression of PDGF and PDGFRA correlated significantly with lymph node metastasis (*p* = 0.010 and *p* = 0.005, respectively; Table 2). High expression of PDGFRB was associated with lymph node metastasis (*p* = 0.012) and lymphovascular invasion (*p* = 0.047).

### 2.3. Serum PDGF-AA and PDGF-BB as Potential Diagnostic Markers 

**Figure 3:**
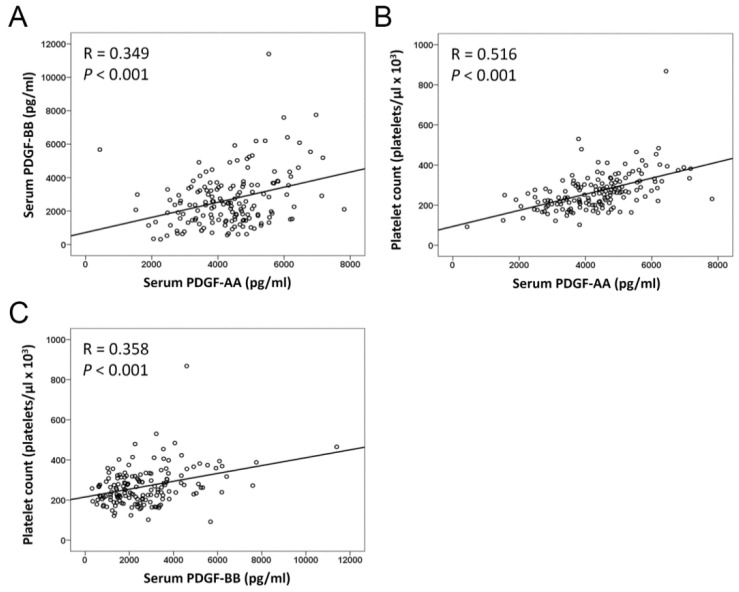
Correlation between preoperative serum PDGF-AA, PDGF-BB, and platelet count. (**A**) Serum PDGF-AA levels are significantly positive correlated with the expression levels of PDGF-BB. (**B**,**C**) Serum PDGF-AA and PDGF-BB correlate positively with platelet count.

Preoperative serum levels of PDGF-AA and PDGF-BB in 146 OSCC patients were measured by ELISA. The mean levels of serum PDGF-AA and PDGF-BB were 4135.0 ± 98.7 pg/mL and 2597.0 ± 132.9 pg/mL, respectively (Table 3). Serum levels of PDGF-AA correlated significantly with lymph node metastasis (*p* = 0.008) and advanced TNM stage (*p* = 0.019; Table 3). In addition, differences were found in the expression of PDGF-BB in lymph node metastasis (*p* = 0.001) and perineural invasion (*p* = 0.007). However, the preoperative serum levels of PDGF-AA and PDGF-BB did not significantly differ among subgroups of OSCC patients defined by age, sex, and lymphovascular invasion. Serum PDGF-AA levels positively correlated with PDGF-BB (R = 0.349, *p* < 0.001). Both serum PDGF-AA and PDGF-BB levels correlated closely with platelet count (R = 0.516, *p* < 0.001 and R = 0.358, *p* < 0.001, respectively; Figure 3).

Furthermore, both serum PDGF-AA and PDGF-BB levels were associated with tumor mRNA level of PDGFA (R = 0.391, *p* = 0.009 and R = 0.475, *p* = 0.001, respectively), PDGFB (R = 0.313, *p* = 0.041, and R = 0.415, *p* = 0.006, respectively), and PDGFRA (R = 0.319, *p* = 0.037 and R = 0.424, *p* = 0.005, respectively; Figure S1). Tumor mRNA level of PDGFRB was significantly associated with serum PDGF-BB (R = 0.395, *p* = 0.009) and marginal significance with serum PDGF-AA (*p* = 0.077; Figure S1D,H).

### 2.4. Thromobocytes Are Elevated in Cervical Lymph Node Metastasis

The mean platelet count in the OSCC group (267.6 ± 7.7 platelet/μL × 10^3^) was significantly higher than in controls (253.2 ± 5.96 platelets/μL × 10^3^, *p* < 0.001). Platelet count was significantly higher in patients with lymph node metastasis (*p* = 0.001) and advanced TNM stage (*p* = 0.019; Table 3). However, platelet count did not differ among the subgroups of patients with OSCC defined by age, gender, T stage, lymphovascular invasion, and perineural invasion.

### 2.5. PDGF and PDGFR mRNA Levels Are Prognostic Values in OSCC Patients

**Figure 4:**
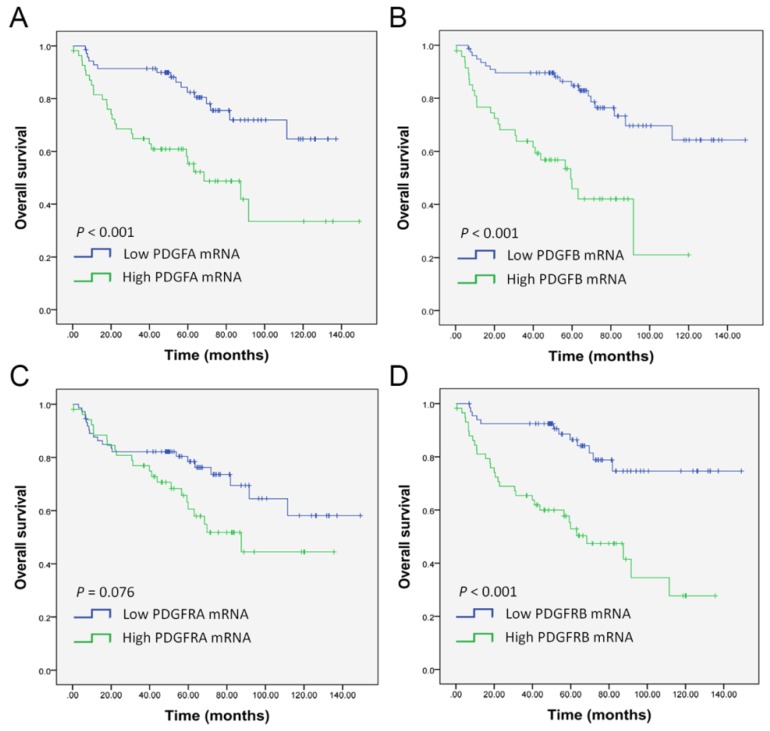
Kaplan–Meier analysis for overall patient survival according to mRNA expression of (**A**) PDGFA, (**B**) PDGFB, (**C**) PDGFRA, and (**D**) PDGFRB in OSCC. *p*-values were calculated by the log-rank test.

A ROC curve analysis was performed to determine the optimal cut-off values for PDGF and PDGFR expression levels and platelet count for predicting overall survival. The cutoff 2-ΔΔCT values were 5.618 (AUC 0.680, 95%CI 0.583–0.777; *p* = 0.001), 4.741 (AUC 0.052, 95%CI 0.544–0.747; *p* = 0.008), 0.911 (AUC 0.543, 95%CI 0.433–0.652; *p* = 0.434), and 6.048 (AUC 0.653, 95%CI 0.553–0.754; *p* = 0.005) for mRNA levels of PDGFA, PDGFB, PDGFRA, and PDGFRB, respectively. Kaplan–Meier analysis revealed that high mRNA levels of PDGFA, PDGFB, and PDGFRB were associated with poor OS in OSCC patients (Figure 4A,B,D). However, no significant differences were found for PDGFRA mRNA expression. In univariate analysis, mRNA levels of PDGFA (HR 2.943, 95%CI 1.581–5.480, *p* = 0.001), PDGFB (HR 3.661, 95%CI 1.967–6.811, *p* < 0.001), and PDGFRB (HR 3.775, 95%CI 1.964–7.253, *p* = 0.001) were significant prognostic factors for OS (Table 4). In the multivariate Cox proportional hazard model, the high mRNA levels of PDGFA, PDGFB, and PDGFRB remained an independent adverse predictor for OS of OSCC compared with those with low expression (adjusted HR for PDGFA 2.798, 95%CI 1.5.1–5.216, *p* = 0.003; adjusted HR for PDGFB 3.935, 95%CI 2.080–7.444, *p* < 0.001; and adjusted HR for PDGFRB 3.496, 95%CI 1.801–6.786, *p* < 0.001, respectively).

**Figure 5:**
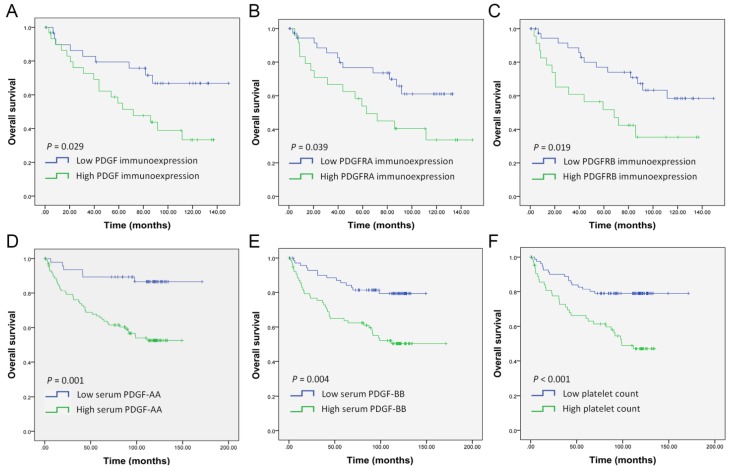
Kaplan–Meier analysis of OSCC patient survival according to predictive gene expression. (**A**–**C**) Estimation of overall survival by immunohistochemical expression of PDGF (**A**), PDGFRA (**B**), and PDGFRB (**C**). (**D**–**F**) Estimation of overall survival by preoperative serum levels of PDGF-AA (**D**), PDGF-BB (**E**), and platelet count (**F**).

Furthermore, we evaluated the possible association between the immunohistochemical expression of PDGF, PDGFRA, and PDGFRB and survival outcome. Kaplan–Meier analysis revealed that high immunoexpression of PDGF (*p* = 0.029), PDGFA (*p* = 0.039), and PDGFRB (*p* = 0.019) were associated with poor OS in OSCC patients (Figure 5). In univariate analysis, high immunoexpression of PDGF (HR 2.828, 95%CI 1.194–6.699, *p* = 0.018) and PDGFRB (HR 2.425, 95%CI 1.130–5.204, *p* = 0.023) were significant prognostic factors for OS; however, no significant differences were found for PDGFRA immunoexpression (Table 4). In multivariate analysis, high immunoexpression of PDGF (HR 2.755, 95%CI 1.161–6.541, *p* = 0.022) and PDGFRB (HR 2.409, 95%CI 1.100–5.275, *p* = 0.028) remained an independent adverse predictor for OS of OSCC compared with those with low expression.

In addition, the cut-off values for preoperative serum PDGF-AA, PDGF-BB, and platelet count were 3619.79 pg/mL (AUC 0.620, 95%CI 0.528–0.712; *p* = 0.016), 2296.04 pg/mL (AUC 0.632, 95%CI 0.542–0.723; *p* = 0.008), and 269.51 platelet/μL × 10^3^ (AUC 0.621, 95%CI 0.522–0.720; *p* = 0.015)*,* respectively. Kaplan–Meier analysis indicated that patients with high serum levels of PDGF-AA (*p* = 0.001), PDGF-BB (*p* = 0.004), and platelet count (*p* < 0.001) had significantly poorer OS than those with low levels (Figure 5D–F). Both univariate and adjusted multivariate Cox regression analyses revealed a poorer OS in patients with high serum levels of PDGF-AA (HR 3.214, 95%CI 1.508–6.851, *p* = 0.002; adjusted HR 2.394, 95%CI 1.112–5.154, *p* = 0.026), PDGF-BB (HR 2.311, 95%CI 1.275–4.188 *p* = 0.006; adjusted HR 2.216, 95%CI 1.218–4.033, *p* = 0.009), and platelet count (HR 2.732, 95%CI 1.533–4.870 *p* = 0.001; adjusted HR 2.207, 95%CI 1.229–3.963, *p* = 0.008; Table 4*)*. 

### 2.6. PDGF Enhances the Tumorigenicity and Metastasis of OSCC Cells in a Dose Dependent Manner

**Figure 6:**
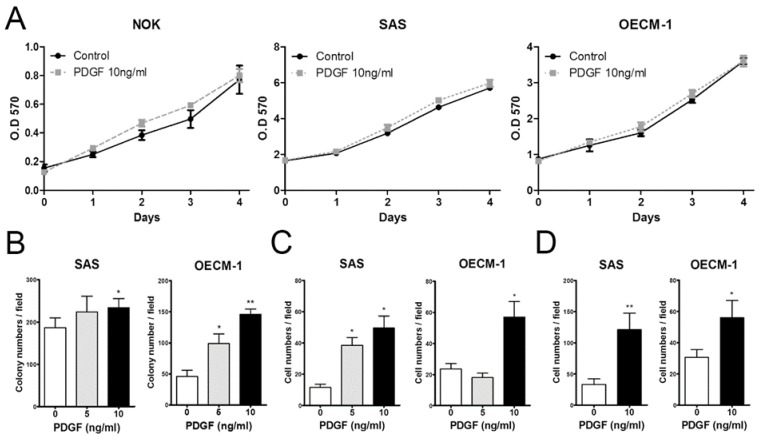
Association between PDGF treatment and oncogenic phenotypes in HNSCC cells. (**A**) The effect of PEGF treatment on NOK, SAS, and OECM-1 cell proliferation was examined. (**B**) Anchorage-independent colony formation; (**C**) migration assay; and (**D**) invasion assay. *, *p* < 0.05; **, *p* < 0.01

To evaluate the effects of PDGF on cultured OSCC cells, we treated SAS and OECM-1 cancer cell lines with two concentrations of PDGF (5 and 10 ng/mL). NOK cells served as controls. Increasing doses of PDGF did not influence the proliferation rate of NOK, SAS, and OECM-1 cells (Figure 6A). However, an elevated exogenous concentration of PDGF was associated with increased colony formation, cell migration, and invasion, relative to PDGF free medium controls in SAS and OECM-1 cells (Figure 6B–D).

## 3. Material and Methods

### 3.1. Patients

The study protocol was approved by the Mackay Memorial Hospital, Taiwan, Institutional Review Board. Patients gave their informed written consent. The diagnosis of OSCC was based on histopathology. CT scans, whole body bone scans, chest radiograms, and whole-abdomen echograms were used for clinical staging. All patients underwent wide tumor excision with modified radical neck dissection as surgical procedure. Samples including the primary tumor and the neck lymph node were collected for histopathological examination. Only OSCC patients without other ailments that might affect their immune response were considered for this study. Patients who had a previous history of malignancy, recent inflammation or any acute infection were not considered. Clinical staging was based on the AJCC TNM stage system, while tumor type and malignancy grade were determined by histopathological analysis. These samples were collected after obtaining written informed consent. This study was approved by the Institutional Review Board (IRB) of Mackay Memorial Hospital, Taipei, IRB project identification code number 15MMHIS104 and 18MMHIS176. 

A total of 149 patients with OSCC were enrolled. Serum samples were collected one day prior to the major operation. All blood samples were drawn by venipuncture after overnight fasting, and none of the cancer patients had received any drug therapy or blood transfusion before the blood collection. Serum was immediately separated by centrifugation at 1000× *g* at 4 °C and stored at −80 °C until subsequent analysis. The platelet count was determined as part of a complete blood count (CBC) with an automated hematology analyzer. Postoperatively, patients were followed for at least 48 months in our department. 

### 3.2. Determination of PDGF in Serum

Preoperative PDGF levels were measured in blood serum with the human Quantikine PDGF-AA and PDGF-BB ELISA Kits (R&D Systems, Minneapolis, MN, USA). These assays use a quantitative sandwich immunoassay. Each serum sample was analyzed in triplicate. Post reaction, the optical density was measured in a spectrophotometer (Thermo Fisher Scientific, Pittsburgh, PA, USA) as directed by the kit’s instructions. Linear calibration curves were obtained with the PDGF standard solutions which came with the kit. 

### 3.3. Quantitative Real-Time Polymerase Chain Reaction (qRT-PCR) of mRNA

Laser capture microdissection was performed to retrieve cells from tumor specimens or non-cancerous matched tissues (NCMT) according to previously established protocols [37]. The miRNeasy Mini Kit (Qiagen, Hilden, Germany) was used to isolate total RNA, which then was reverse transcribed into cDNA. TaqMan gene expression assays (Applied Biosystems, Foster City, CA, USA) were used to quantify the mRNA expression of PDGFA (Assay ID Hs00964426), PDGFB (Assay ID Hs00966522), PDGFRA (Assay ID Hs00998018), PDGFRB (Assay ID Hs01019589), and GAPDH as internal control (Assay ID Hs99999905) according to the manufacturer’s instructions. The threshold cycle (Ct) method was used to measure relative changes in expression. Data were analyzed using the –ΔΔCt method and the abundance of PDGF and PDGFR mRNA was calculated relative to the internal controls. The relative mRNA expression levels were calculated using the 2^−ΔΔCt^ method.

### 3.4. Immunohistochemistry

PDGF, PDGFRA, and PDGFRB immunoreactivity was detected by immunohistochemical analyses according to previously reported protocols [38]. Slides were stained with primary antibodies for PDGF (PU376; BioGenex, San Ramon, CA, USA; diluted 1:200), PDGFRA (sc-338; Santa Cruz, CA, US; diluted 1:200), and PDGFRB (sc-339; Santa Cruz; diluted 1:200). Preimmune rabbit IgG served as negative control. The intensity of immunoreactivity was scored in four categories: 0 (no staining), 1+ (weak staining) 2+ (moderate staining), and 3+ (strong staining). Scores of 2+ and 3+ were classified as positive staining. Slides showing ≥50% positive cancer cells were classified as having high expression, and those with <50% positive cancer cells were considered to have low expression.

### 3.5. Cell Culture, Reagents, and Phenotypic Assays

The OSCC cell lines SAS, OECM-1, and normal human oral keratinocytes (NOK) cells were cultured as previously described [38]. PDGF-conditioned medium (5 and 10 ng/mL) was freshly prepared from PDGF (Sigma-Aldrich, St Louis, MO, USA) in growth medium. The phenotype including cell viability, proliferation, migration, and invasion was analyzed.

### 3.6. Cell Proliferation Assays

A total of 3000 cells were seeded in culture dishes to grow for various time periods. Trypan blue exclusion assays (Sigma-Aldrich) were used to evaluate cell viability. Cell number was plotted as a function of time in culture. 

### 3.7. Anchorage-Independent Colony Formation

Cells were suspended in 1.3% methylcellulose (Sigma-Aldrich), plated on a layer of 0.9% agarose (Sigma-Aldrich) in culture media containing 15% Fetal Bovine Serum (FBS, Biotechnology, Industries, Tel Aviv, Israel) and then cultured at 37 °C for 7 days. The colonies were washed twice with PBS, fixed with methanol and stained with 0.05% crystal violet. Colonies with a diameter >50 μm in more than five fields per well were counted in triplicate experiments [39].

### 3.8. Transwell Migration and Invasion Assay

Cells were grown in media containing 0.5% FBS on transwell membranes (Corning, Acton, MA, USA) with a pore diameter of 8 µm. For the migration assay, cells were seeded into the upper chamber of a transwell at a density of 1 × 10^5^ cells per well. For invasion assay, the transwell membrane was coated with Matrigel basement membrane matrix (BD Biosciences, Fairleigh, NJ, USA) and then 2 × 10^5^ cells were seeded onto the Matrigel coated transwell. After 24 h, the migrating or invading cells on the lower surface of the membrane were stained with Hoechst 33258 (Sigma-Aldrich) and counted under a fluorescence microscope.

### 3.9. Statistics 

The data was analyzed with SPSS 17.0 (SPSS Inc., Chicago, IL, USA) and is presented as mean ± standard error of the mean. The expression levels of PDGFA, PDGFB, PDGFRA, and PDGFRB mRNA in OSCC as well as in the control tissue were evaluated. The differences of PDGFA, PDGFB, PDGFRA, and PDGFRB mRNA expression levels between the two groups were analyzed with a comparative 2^−ΔΔCt^ method and Student’s *t*-test. In addition, immunohistochemistry was employed to detect immunoexpression of PDGF, PDGFRA, and PDGFRB protein. A cross-comparison of immunoexpression and clinical pathological parameters was performed with a chi-squared test for correlation. Student’s *t*-test was conducted to statistically analyze the correlation between serum PDGF-AA, PDGF-BB, and platelet count and the clinical-pathological parameters of the patients. By using receiver operating characteristic (ROC) analysis, different clinical subsets could be efficiently separated by the obtained levels; the area under the curve (AUC) was used to test for discriminative ability. Kaplan–Meier analysis was used to calculate disease specific overall survival. Finally, Cox’s proportional hazards regression model was used for multivariate logistic regression analysis to identify independent prognostic factors. Differences were considered to be statistically significant at any of the following conditions: * *p* < 0.05, ** *p* < 0.01, *** *p* < 0.001. Cross-comparisons with no significance were not marked.

## 4. Discussion

The transformation from normal oral squamous cells to oral cancer is a multistep process. Numerous genetic mutations are required to accumulate in the process, which is possibly caused by the consumption of areca (betel) nuts, tobacco, alcohol, or by viruses. PDGF facilitates the hyperplasia of vessel endothelial cells and attracts the infiltration of nearby vessel cells, resulting in angiogenesis [22]. Hellstrom and Kalen indicated that the PDGFB secreted by the endothelium facilitates the proliferation and migration of vascular smooth muscle cells, leading to vessel enlargement and angiogenic sprouting [40]. PDGF is a critical factor for tumor growth, angiogenesis, and tumor survival. It is the main control factor in the transition from the G0 to the G1 phase of the cell cycle [41]. The PDGFRA/JNK-1 pathway is critical in the control of apoptosis because this signaling pathway not only influences apoptosis but also fosters p21 expression and inhibits the transition from the G1 to the S phase of the cell cycle [42]. The different types of PDGFR are inducing different downstream responses; PDGFRA and PDGFRB facilitate cell growth [43], angiogenesis [44], and chemotaxis [45]. PDGFRB inhibits apoptosis and PDGFRA/B can promote cell division [46,47]. However, the knowledge about the roles of PDGFC and PDGFD signaling and the respective cellular responses remain fairly limited. Bran et al. are reporting the expression of PDGF-AA in different types of head and neck squamous cell carcinoma cells but no expression of PDGF-BB. The concentrations of PDGF-AA and BB in the serum of 88 patients with cancer were higher than in the control group [48]. Aebersold et al. found that 54% of the tumor samples from 95 patients with oropharyngeal cancer were positively stained for PDGF-BB, which correlated with their risk of cancer metastasis [49]. 

In this study we investigated PDGF and PDGFR more comprehensively. We found that the expression of PDGFA and PDGFB mRNA was higher in cancerous tissue than in the adjacent normal tissue. In addition, PDGFRB was overexpressed in cancerous and metastatic lymph node tissue. PDGFB binds to PDGFRB and is speculated to play a crucial role in the tumorigenesis of oropharyngeal cancer. Furthermore, PDGFRB facilitates cell hyperplasia and angiogenesis, but the actual mechanism and the downstream effectors involved require further investigation. Subsequently, through immunohistochemical analysis with tissue staining and comparison with clinicopathological parameters, we found that high expression of PDGF and PDGFRA correlated significantly with lymph node metastasis. Elevated expression of PDGFRB was associated with lymph node metastasis and lymphovascular invasion. This might be caused by the tumor-produced PDGFB which promotes the disassociation of vascular smooth muscle cells (VSMCs) from the tumor vasculature. This finding contradicts the known effect of PDGFB on the recruitment of VSMCs into the newly formed blood vessels [50,51]. 

In addition, the serum levels of PDGF isoforms AA and BB were increased in OSCC patients. A correlation analysis of PDGF and clinicopathological parameters also was performed, and an elevated expression of PDGF-AA was found to increase with lymph metastasis and late staging. PDGF-BB correlated with lymph metastasis and perineural invasion. Furthermore, the disease specific survival rates of patients with higher levels of PDGF-AA and BB decreased. Therefore, the expression of PDGF is playing a crucial role in the genesis, metastasis, and prognosis of OSCC. In colon cancer [52,53] and in lung cancer [54], PDGF has been associated with poor survival, like we found for OSCC. The reason for this may be that the elevated serum levels of PDGF before surgery go along with the tumor burden and with metastasis in the neck lymph nodes, which both have an adverse effect on patients with OSCC. However, the detailed mechanisms and the real reasons for this correlation still need to be investigated. 

The clinical results of some studies on esophageal and stomach cancer show that higher platelet counts are related to late tumor staging and reduced survival rates [13,14]. Other studies related to thrombocythemia have found that, compared to people with normal amounts of platelets, apart from lower survival rates in various types of cancer such as breast, lung, kidney, and colorectal cancers [15,55,56], patients with thrombocythemia are more likely to experience thrombosis as complication, contributing to a higher mortality rate. Therefore, having thrombocytosis may be considered as an independent indicator for the poor prognosis of malignant tumors. Lu et al. found that patients with oral cancer had significantly increased platelet counts, particularly those with larger tumors and lymph node metastasis. Moreover, the increase in platelets was mostly found in patients with late-stage oral cancer, relapsing tumors, and metastasis [16]. In addition, patients with preoperative thrombocytosis had a significantly decreased total postoperative survival rate [16]. Thus, a series of cytokines are produced, which stimulate the migration of white blood cells and blood vessel endothelial cells. The cytokines themselves also induce influencing factors such as PDGF, which facilitates epithelialization and angiogenesis, thereby helping the growth, invasion, and migration of tumors. Furthermore, studies on colorectal cancer have found an apparent increase in preoperative platelets as well as increased PDGF in serum. In addition, PDGF was found to be related to thrombocytosis [57]. Our study revealed positive correlations between PDGF-AA, BB, and platelet count. These correlations indirectly indicate that platelets may secrete PDGF in addition to tumors and contribute to their rapid growth and metastasis. In another study of CRC patients, the levels of PDGF-AB and sP-selectin were multiples of the normal values, whereas the platelet count was lower than in the control group. However, no positive correlation was found between the platelet count and the PDGF-AB and sP-selectin levels [58]. In thyroid cancer, the BRAF T1799A mutation is associated with aggressive pathological outcomes of PTC in which high platelet counts and increased PDGF production are observed [59]. The expression level of PDGFB and PDGFRB in the bone marrow of essential thrombocythemia patients were significantly higher than in normal controls [60]. Strong positive correlations of multiple serum cytokines (most notably IL-7, IL-1RA, and PDGFB) with platelet count have been observed. Possible explanations are (a) that platelets store and release these cytokines or that platelets contribute to their production, (b) these cytokines enhance the production of platelets, or (c) other, yet unrecognized causes, like e.g., shared background factors, are playing a role [61]. Our data show strong positive correlations between platelet count and PDGF-AA and BB. Platelet count is independent to the tumor size, indicating that the increased serum levels of PDGF-AA and BB might originate from platelets besides from the neoplasms.

The crucial role of PDGF in OSCC growth and the observed clinical relationship strongly suggests a correlation between the PDGF/PDGFR pathway networks and OSCC [49]. Dysfunction of PDGF signaling and the overexpression of the PDGFRs have been found in several pathological conditions of OSCC. Based on these findings, it was suggested to target PDGFR in the treatment of OSCC. Although the roles of PDGF and PDGFR in angiogenesis and their possible molecular mechanisms have not yet been fully understood to date, tyrosine kinase inhibitors were shown to reduce angiogenesis and tumor growth in experimental models using xenografts. Moreover, they recently have been demonstrated to be effective in chemotherapy resistant tumors [49,62]. Targeting the autophosphorylation of PDGFR with receptor tyrosine kinases inhibitors therefore may be a promising strategy for future tumor therapy by autocrine and paracrine inhibition of tumor growth and angiogenesis, presumably through simultaneous downregulation of PDGF.

## 5. Conclusions 

In patients with OSCC, elevated levels of serum PDGF and platelet count are associated with neck lymph node metastasis, advanced TNM stage, and poor survival. This suggests that the preoperative level of serum PDGF and thrombocythemia should be considered as a prognostic biomarker. Overexpression of PDGFRA and PDGFRB is associated with lymph node metastasis and poor prognosis. PDGFRB is associated with lymphovascular invasion. This suggests that PDGFR should be considered as a potential new therapeutic target for OSCC. 

## Figures and Tables

**Table 1:** Relationship between clinical parameters and mRNA expression of PDGF and PDGFR in OSCC patients.

Variables	N	PDGFA		PDGFB		PDGFRA		PDGFRB	
		Mean ± SEM	*p*-Value	Mean ± SEM	*p*-Value	Mean ± SE	*p*-Value	Mean ± SEM	*p*-Value
Gender									
Male	117	9.63 ± 1.02	0.124	6.37 ± 0.77	0.502	3.25 ± 0.62	0.303	10.99 ± 1.29	0.615
Female	9	16.81 ± 10.59		8.29 ± 3.87		0.93 ± 0.34		13.47 ± 5.63	
Age									
< 54	70	9.95 ± 1.30	0.858	7.46 ± 1.13	0.147	3.29 ± 0.91	0.696	11.55 ± 1.65	0.740
≥ 54	56	10.38 ± 2.17		5.32 ± 0.83		2.83 ± 0.64		10.70 ± 1.96	
T stage									
T1-2	32	8.54 ± 2.12	0.440	5.70 ± 1.29	0.521	3.23 ± 1.06	0.884	9.93 ± 2.42	0.567
T3-4	94	10.68 ± 1.44		6.78 ± 0.88		3.03 ± 0.69		11.59 ± 1.48	
N status									
N0	80	7.34 ± 1.07	0.002 **	5.10 ± 0.90	0.011 *	2.27 ± 0.42	0.064	9.05 ± 1.50	0.026 *
N+	46	15.00 ± 2.57		8.95 ± 1.17		4.49 ± 1.39		14.85 ± 2.17	
Stage									
I-II	21	6.41 ± 1.79	0.165	3.33 ± 0.81	0.051	1.39 ± 0.51	0.192	5.51 ± 2.45	0.045 *
III-IV	105	10.89 ± 1.39		7.14 ± 0.85		3.42 ± 0.68		12.30 ± 1.41	
Lymphovascular invasion									
No	108	9.48 ± 1.28	0.185	6.33 ± 0.08	0.557	2.46 ± 0.43	0.445	10.31 ± 1.28	0.078
Yes	18	14.04 ± 3.37		7.57 ± 1.80		3.41 ± 1.62		16.32 ± 3.12	
Perineural invasion									
No	97	9.85 ± 1.46	0.664	6.01 ± 0.81	0.216	2.84 ± 0.48	0.438	10.22 ± 1.32	0.169
Yes	29	11.10 ± 1.85		8.16 ± 1.67		3.91 ± 1.95		14.35 ± 3.21	

*, *p* < 0.05; **, *p* < 0.01.

**Table 2:** Relationship between clinical parameters and immunoexpression of PDGF and PDGFR in OSCC patients.

Variables	N	PDGF			PDGFRA			PDGFRB		
		Low	High	*p*-Value	Low	High	*p*-Value	Low	High	*p*-Value
Gender										
Male	57	25 (43.9%)	32 (56.1%)	0.773	33 (57.9%)	24 (42.1%)	0.710	35 (61.4%)	22 (38.6%)	0.184
Female	6	3 (50.0%)	3 (50.0%)		3 (50.0%)	3 (50.0%)		2 (33.3%)	4 (66.7%)	
Age										
<54	35	17 (48.6%)	18 (51.4%)	0.461	21 (60.0%)	14 (40.0%)	0.608	23 (65.7%)	12 (34.3%)	0.208
≥54	28	11 (39.9%)	17 (60.7%)		15 (53.6%)	13 (46.4%)		14 (50.0%)	14 (50.0%)	
T stage										
T1-2	14	8 (57.1%)	6 (42.9%)	0.278	10 (71.4%)	4 (28.6%)	0.221	9 (64.3%)	5 (35.7%)	0.632
T3-4	49	20 (40.8%)	29 (59.2%)		26 (53.1%)	23 (46.9%)		28 (57.1%)	21 (42.9%)	
N status										
N0	36	21 (58.3%)	15 (41.7%)	0.010 *	26 (72.2%)	10 (27.8%)	0.005 **	26 (72.2%)	10 (27.8%)	0.012 *
N+	27	7 (25.9%)	20 (74.1%)		10 (37.0%)	17 (63.0%)		11 (40.7%)	16 (59.3%)	
Stage										
I-II	7	4 (57.1%)	3 (42.9%)	0.473	5 (71.4%)	2 (28.6%)	0.418	5 (71.4%)	2 (28.6%)	0.469
III-IV	56	24 (42.9%)	32 (57.1%)		31 (55.4%)	25 (44.6%)		32 (57.1%)	24 (42.9%)	
Lymphovascular invasion										
No	51	24 (47.1%)	27 (52.9%)	0.389	31 (60.8%)	20 (39.2%)	0.229	33 (64.7%)	18 (35.3%)	0.047 *
Yes	12	4 (33.3%)	8 (66.7%)		5 (41.7%)	7 (58.3%)		4 (33.3%)	8 (66.7%)	
Perineural invasion										
No	54	24 (44.4%)	30 (55.6%)	0.535	31 (60.8%)	5 (39.2%)	0.917	34 (63.0%)	20 (37.0%)	0.293
Yes	9	5 (55.6%)	4 (44.4%)		5 (55.6%)	4 (44.4%)		4 (44.4%)	5 (55.6%)	

*, *p* < 0.05; **, *p* < 0.01.

**Table 3:** Relationship between clinical parameters and preoperative serum PDGF-AA, PDGF-BB, and platelet count in OSCC patients.

Variables	N	PDGF-AA (pg/mL)		PDGF-BB (pg/mL)		Platelet Counts (Platelets/μL × 10^3^)	
		Mean ± SEM	*p*-Value	Mean ± SEM	*p*-Value	Mean ± SEM	*p*-Value
Gender							
Male	129	4180.2 ± 107.2	0.247	2611.3 ± 148.5	0.789	267.9 ± 8.3	0.912
Female	20	3843.9 ± 244.6		2506.5 ± 257.2		265.5 ± 20.9	
Age							
<54	73	4013.3 ± 143.3	0.228	2702.3 ± 191.8	0.440	270.8 ± 12.8	0.684
≥54	76	4252.0 ± 135.4		2496.3 ± 184.7		264.5 ± 8.8	
T stage							
T1-2	67	3955.3 ± 136.6	0.100	2445.7 ± 192.9	0.304	259.0 ± 10.3	0.316
T3-4	82	4281.9 ± 138.9		2721.0 ± 182.8		274.6 ± 11.2	
N status							
N0	107	3971.0 ± 106.4	0.008 **	2323.5 ± 124.9	0.001 **	251.8 ± 89.9	0.001 **
N+	42	4553.1 ± 210.1		3294.6 ± 326.6		307.8 ± 93.3	
Stage							
I-II	51	3816.0 ± 141.1	0.019*	2346.0 ± 188.0	0.173	242.7 ± 10.6	0.019 *
III-IV	98	4301.1 ± 128.1		2727.9 ± 175.9		280.6 ± 10.1	
Lymphovascular invasion							
No	130	4083.4 ± 101.6	0.172	2592.8 ± 145.5	0.932	264.1 ± 8.1	0.236
Yes	19	4488.4 ± 336.9		2627.2 ± 316.9		291.5 ± 23.7	
Perineural invasion							
No	130	4094.5 ± 100.9	0.284	2461.5 ± 135.7	0.007 **	267.7 ± 8.3	0.963
Yes	19	4412.5 ± 350.9		3525.7 ± 424.6		266.7 ± 20.9	

*, *p* < 0.05; **, *p* < 0.01.

**Table 4:** Univariate and multivariate analysis of risk factors for overall survival.

		Univariate Analysis		Multivariate Analysis	
Variables	Subgroups	HR (95%CI)	*p*-Value	Adjusted HR (95%CI)	*p*-Value
PDGFA mRNA	High vs. Low	2.943 (1.581−5.480)	0.001 **	2.798 (1.501−5.216)	0.003 *
PDGFB mRNA	High vs. Low	3.661 (1.967−6.811)	<0.001 ***	3.935 (2.080−7.444)	<0.001 ***
PDGFRA mRNA	High vs. Low	1.711 (0.938−3.122)	0.080	1.550 (0.846−2.838)	0.156
PDGFRB mRNA	High vs. Low	3.775 (1.964−7.253)	0.001 **	3.496 (1.801−6.786)	<0.001 ***
PDGF immunexpression	High vs. Low	2.828 (1.194−6.699)	0.018 *	2.755 (1.161−6.541)	0.022 *
PDGFRA immunexpression	High vs. Low	2.108 (0.977−4.548)	0.057	2.020 (0.931−4.383)	0.075
PDGFRB immunexpression	High vs. Low	2.425 (1.130−5.204)	0.023 *	2.409 (1.100−5.275)	0.028 *
Serum PDGF-AA	High vs. Low	3.214 (1.508−6.851)	0.002 **	2.394 (1.112−5.154)	0.026 *
Serum PDGF-BB	High vs. Low	2.311 (1.275−4.188)	0.006 **	2.216 (1.218−4.033)	0.009 **
Platelet count	High vs. Low	2.732 (1.533−4.870)	0.001 **	2.207 (1.229−3.963)	0.008 **

HR, hazard ratio; CI, confidence interval; Adjusted for age, gender, and TNM stage; *, *p* < 0.05; **, *p* < 0.01; ***, *p* < 0.001.

## Supplementary Materials

Supplementary materials can be found at https://www.mdpi.com/1422-0067/21/7/2360/s1. Figure S1. Correlation between tumor mRNA expression profile and preoperative serum protein level.
