# Supplementary material for: Overexpression of Platelet-Derived Growth Factor and Its Receptor Are Correlated with Oral Tumorigenesis and Poor Prognosis in Oral Squamous Cell Carcinoma
Source: Int J Mol Sci. 2020 Mar 29;21(7):2360. doi: 10.3390/ijms21072360 (PMC7177415; doi:10.3390/ijms21072360)
Supplement: Supplementary file 1 [file ijms-21-02360-s001.zip › ijms-747748-supp-revise/ijms-747748-supp-revisepdf.pdf]

Figure S1. Correlation between tumor mRNA expression profile and preoperative serum protein level. The correlation of the serum PDGF-AA level with the mRNA expression of PDGFA (A), PDGFB (B), PDGFRA (C), and PDGFRB (D) were assessed. Serum PDGF-BB levels was correlated with the mRNA levels of PDGFA (E), PDGFB (F), PDGFRA (G), and PDGFRB (H).
