# Supplementary figures and images for: Overexpression of Platelet-Derived Growth Factor and Its Receptor Are Correlated with Oral Tumorigenesis and Poor Prognosis in Oral Squamous Cell Carcinoma
Source: Int J Mol Sci. 2020 Mar 29;21(7):2360. doi: 10.3390/ijms21072360 (PMC7177415; doi:10.3390/ijms21072360)

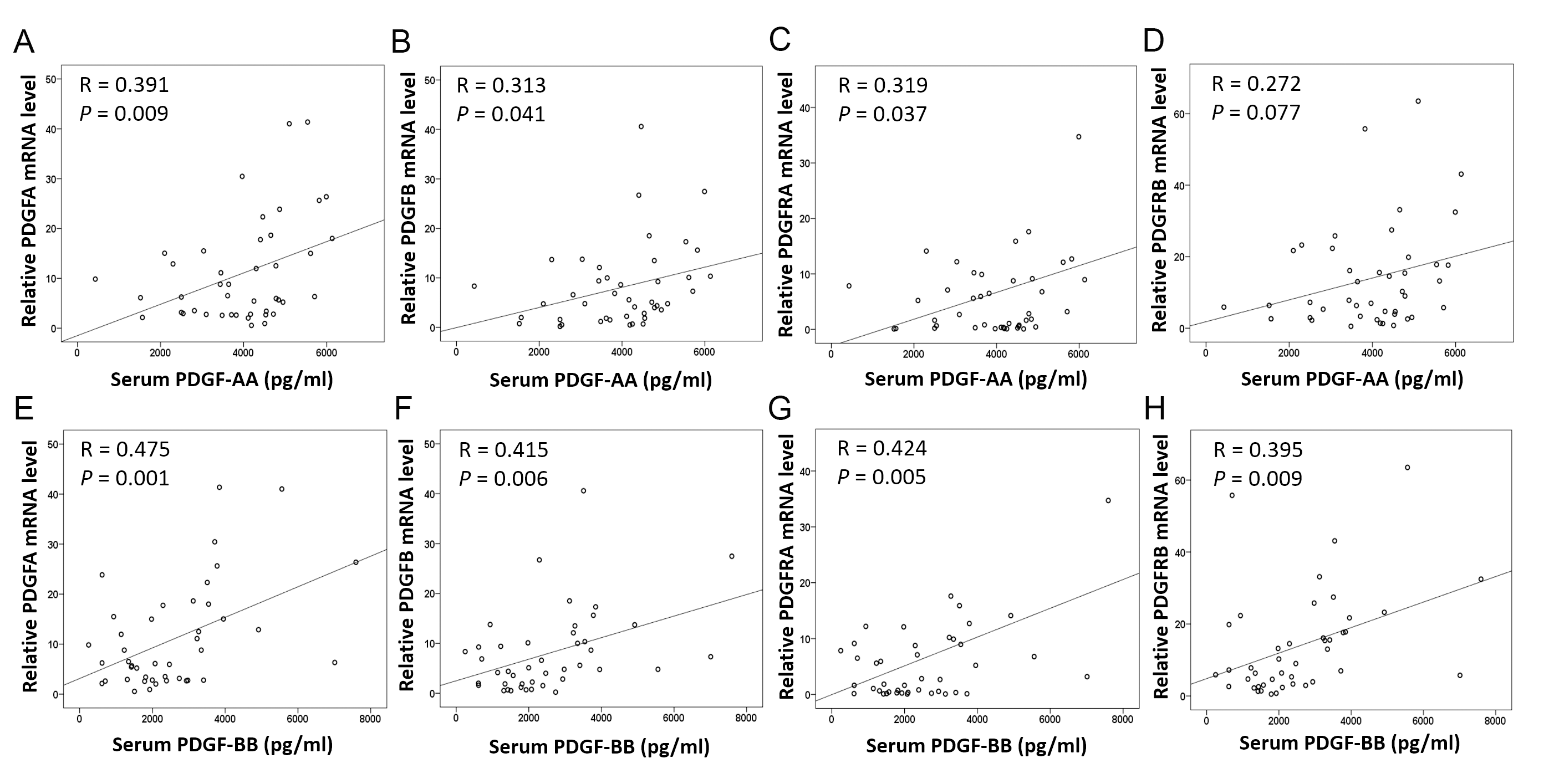

Supplement: Supplementary file 1 [file ijms-21-02360-s001.zip › ijms-747748-supp-revise/ijms-747748-supp-revise.tif]
